# A Study of a PID Controller Used in a Micro-Electrical Discharge Machining System to Prepare TiO_2_ Nanocolloids

**DOI:** 10.3390/nano10061044

**Published:** 2020-05-29

**Authors:** Kuo-Hsiung Tseng, Yur-Shan Lin, Chaur-Yang Chang, Meng-Yun Chung

**Affiliations:** Department of Electrical Engineering, National Taipei University of Technology, Taipei 10608, Taiwan; linyurshan@gmail.com (Y.-S.L.); cychang@ntut.edu.tw (C.-Y.C.); alexmychung@gmail.com (M.-Y.C.)

**Keywords:** electric spark discharge method, TiO_2_ nanocolloids, Ziegler–Nichols method, PID controller, electrical discharge machining

## Abstract

This study developed a micro-electrical discharge machining (micro-EDM) system for producing TiO_2_ nanocolloids. When a proportional–integral–derivative controller designed using the Ziegler–Nichols method was adopted to control the interelectrode gap, TiO_2_ nanocolloids were obtained from spark discharges generated between two titanium wires immersed in deionized water. For a pulse on time–off time of 40–40 μs and a colloid production time of 100 min, TiO_2_ nanocolloids were produced that had an absorbance of 1.511 at a wavelength of 245 nm and a ζ potential of −47.2 mV. They had an average particle diameter of 137.2 nm, and 64.2% of particles were smaller than 91.28 nm. The minimum particles were spherical. The characteristics of colloids confirmed that the micro-EDM system can produce TiO_2_ nanocolloids with excellent suspension stability. The colloid production method proposed in this study has the advantages of low equipment cost and no dust diffusion in the process environment. These advantages can improve the competitiveness of the electric spark discharge method for high-quality TiO_2_ nanoparticle production. The colloids produced in this study did not contain elements other than titanium and oxygen, and they may prevent secondary environmental pollution.

## 1. Introduction

The range of applications of titanium dioxide (TiO_2_) nanoparticles (NPs) has recently been extended to personal protective products [1,2], biomedical materials [3], environmental pollution prevention [4,5,6], and anticancer care [7]. Therefore, the TiO_2_ NP production method has become a crucial part of nanotechnology. Because TiO_2_ nanocolloids produced using chemical methods contain elements other than titanium and oxygen due to the suspending agent added during the process [8,9,10,11], these TiO_2_ nanocolloids can only be employed in certain fields. In addition, dust diffusion during the colloid production process is harmful to the human body and environment [12].

**Figure 1:**
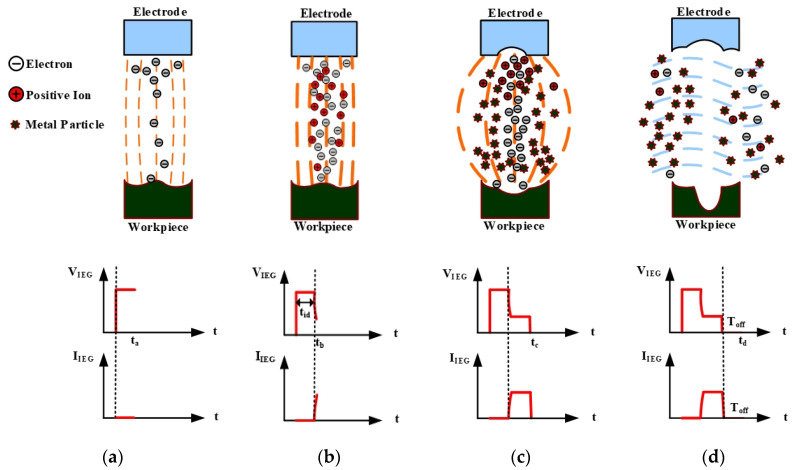
Process of electrical discharge machining (EDM): (**a**) the discharge, (**b**) ionization, (**c**) slag generation and expulsion, and (**d**) insulation recovery of dielectric fluid.

The electric spark discharge method (ESDM) for producing TiO_2_ nanocolloids can solve the aforementioned shortcomings of using chemical methods for producing TiO_2_ NPs. The principle of producing TiO_2_ NPs by using the ESDM is to use the high temperature generated by electric sparks to evaporate the surface of Ti metal wires immersed in deionized water (DW) [13]. The evaporated steam is rapidly cooled by DW and condensed into TiO_2_ NPs. The crystal structure of the sample prepares by ESDM was measured by XRD, which contained both titanium oxide and Ti simultaneously [14]. Figure 1 shows the ESDM process [15]. Figure 1a illustrates the occurrence of electric spark discharge. The electric-field strength of the interelectrode gap (IEG) is higher when the distance between electrode and workpiece is smaller. When the electric-field gradient is greater than the insulation withstanding voltage of the dielectric fluid, a discharge channel is established in the IEG, and a tiny number of electrons are emitted from the electrode surface toward the workpiece [16]. Figure 1b shows ionization. Electrons ejected from the electrode to the workpiece rapidly collide with neutral atoms in the dielectric fluid to form free electrons and positive ions because of acceleration of the electric field. Free electrons and positive ions are attracted to the opposite electrode, forming a narrow plasma column, at which point the IEG current rises rapidly. Figure 1c illustrates the slag generation and expulsion. Ionization occurs again at this stage, and generated electrons and positive ions continuously collide with the surface of the electrode and workpiece. At this instant, high kinetic energy is converted into considerable heat energy, which causes the impacted surface material to melt and evaporate. The dielectric fluid vaporizes and expands because of the high temperature, and molten material is expelled from the IEG. The dielectric breakdown causes $I_{\mathrm{IEG}}$ to increase to the limit value, and $V_{\mathrm{IEG}}$ decreases as the resistance of the dielectric fluid decreases. Figure 1d shows the insulation recovery of the dielectric fluid. When T_off_ is reached, the power to the electrode and workpiece is turned off, and the electrode ceases to release electrons. $I_{\mathrm{IEG}}$ decreases accordingly, and the dielectric fluid reenters its insulation state. At this stage, $I_{\mathrm{IEG}}$ and $V_{\mathrm{IEG}}$ both fall to zero. Metal particles and positive ions produced through melting are suspended in the dielectric fluid. Nanocolloids can be produced by repeating this discharge process. In Figure 1, after the pulse voltage appears, an insulation delay time (ignition delay time, t_id_) will pass before the insulation of the dielectric fluid collapses. The accumulation time of t_id_ will increase as the frequency of the pulse voltage increases [17]. When the duty cycle of the discharge pulse is 50%, the T_off_ period of the small pulse period is enough to restore the electrode gap to the insulated state. At this time, the t_id_ time accumulated in the process can be reduced by reducing the frequency of the pulse voltage and to improve the absorption of the colloid. During the T_off_ period of a large pulse period, the electrode gap will be too late to restore the insulation of the electrode gap due to the excessive energy remaining in the electrode gap. This characteristic will lead to the failure of the next discharge cycle and the reduction in the discharge success rate of the electrode gap. With the previous result, the absorption of the colloid will decrease. Therefore, discussing suitable T_on_–T_off_ to improve the absorption of colloids is one of the topics of the nano colloid prepared by ESDM.

However, the industrial-grade electrical discharge machines used in the ESDM are costly [18,19,20]. In this study, a micro- electrical discharge machining (EDM) system was developed to reduce the cost of fabricating TiO_2_ nanocolloids by using the ESDM. The integral of the time-weighted absolute error (ITAE) performance criterion was used to evaluate the control exerted by the IEG, and the performance of the system for producing colloids was analyzed using relevant instruments.

## 2. Materials and Methods

### 2.1. The Micro-EDM System

**Figure 2:**
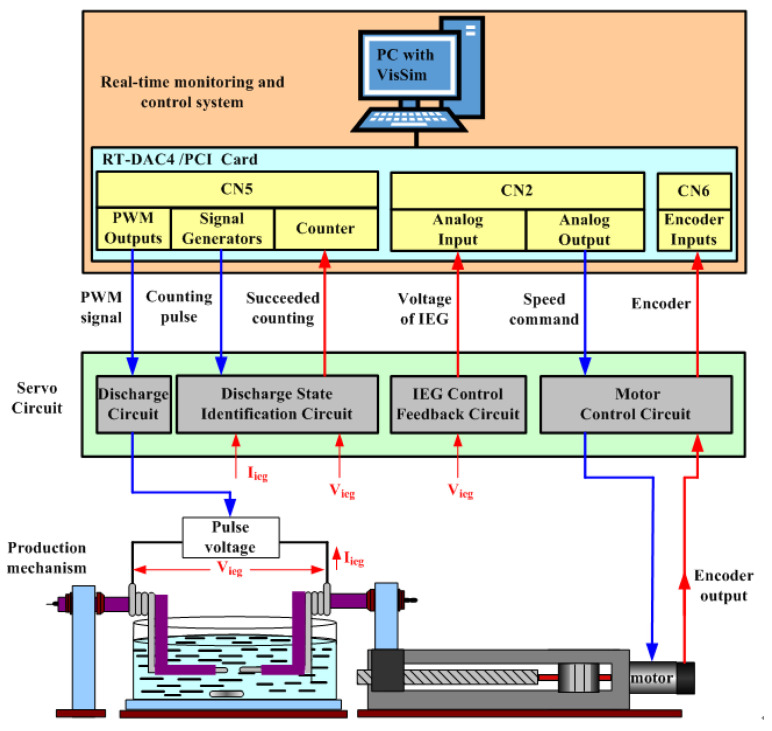
Architecture of micro-EDM system.

This study developed a micro-EDM system with real-time monitoring and control functions for producing TiO_2_ nanocolloids on the basis of the ESDM. This system consists of a production mechanism, servo circuit, and a real-time monitoring and control (RTMAC) system, and its architecture is displayed in Figure 2. The RTMAC system comprises a personal computer, the VisSim software, and an RT-DAC4/PCI card [21,22]. The function of the RT-DAC4/PCI card is to process the output–input signal between VisSim and the servo circuit. The cost of the micro-EDM system is much lower than that of industrial-grade electrical discharge machines and costs approximately USD 5300.

**Figure 3:**
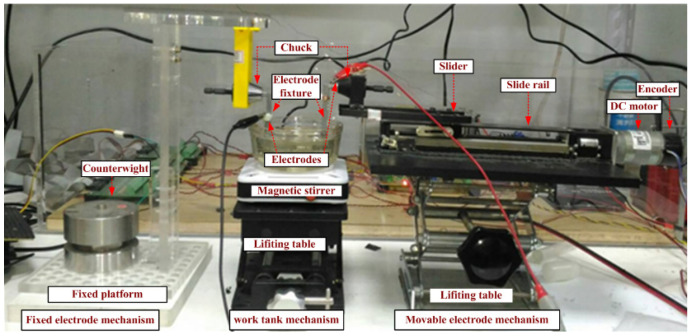
Production mechanism in the micro-EDM system.

Figure 3 illustrates the production mechanism. Electrodes in the fixed electrode mechanism are immovable. The position of the slider on the slide rail is controlled by a motor and changes the position of electrodes in the movable electrode mechanism. Therefore, IEG control is exerted through the closed-loop control of the DC motor. The function of the optical encoder is to provide the moving distance of the movable electrode, whereas that of the magnetic stirrer in the work tank mechanism is to uniformly disperse the produced NPs in the dielectric liquid.

The functions of each part of the servo circuit are as follows:

1. Discharge circuit: The IEG pulse voltage is provided by controlling the on/off state of the transistor [23,24].
2. Motor control circuit: IEG control and IEG displacement measurement are performed [25,26].
3. IEG control feedback circuit: the $V_{\mathrm{IEG}}\;$ signal required for closed-loop control of the IEG is captured.
4. Discharge state identification circuit: this circuit is used to determine the discharge state and provide the signal required to calculate the discharge success rate (DSR).

### 2.2. Proportional–Integral–Derivative (PID) Controller of the IEG and the ITAE Performance Criterion

This study used the root locus and Ziegler–Nichols (Z–N) methods to design a PID controller for motor speed control and evaluated the effectiveness of IEG servo control by calculating the ITAE performance criterion [27,28].

#### 2.2.1. Design of the PID Controller by Using the Root Locus Method

Certain studies have employed the root locus method to design a PID controller of IEG and have obtained PID controller parameters through the transfer function of the motor. Equation (1) shows the DC motor transfer function, where *θ_m_* is the rotor position; *V_a_* is the armature voltage; *R_a_* and *L_a_* are the resistance and inductance of the armature coil, respectively; and *J_m_*, *B_m_*, *K_e_*, and *K_t_* are the moment of inertia, viscous friction coefficient of the motor, back electromotive force constant, and torque constant, respectively. Equation (1) can be rewritten as Equation (2) when *n*_0_ = *K_t_*, *d*_1_ = *B_m_R_a_* + *K_t_K_e_*, *d*_2_ = *J_m_R_a_* + *B_m_L_a_* and *d*_3_ = *J_m_L_a_*.

(1) $$\frac{\theta_{m}\left(s\right)}{V_{a}\left(s\right)}=\frac{K_{t}}{J_{m}L_{a}s^{3}+\left(J_{m}R_{a}+B_{m}L_{a}\right)s^{2}+\left(B_{m}R_{a}+K_{t}K_{e}\right)s}$$

(2) $$G_{p}\left(s\right)=\frac{\theta_{m}\left(s\right)}{V_{a}\left(s\right)}=\frac{n_{0}}{d_{3}s^{3}+d_{2}s^{2}+d_{1}s}$$

When proportional controller *K′* is added to Equation (2), the closed-loop transfer function *T*(*s*) can be expressed as Equation (3), and the characteristic equation can be expressed as Equation (4). Table 1 shows a Routh table established on the basis of the characteristic equation.

(3) $$T\left(s\right)=\frac{K^{\prime }G_{p}\left(s\right)}{1+K^{\prime }G_{p}\left(s\right)}=\frac{n_{0}K^{\prime }}{d_{3}s^{3}+d_{2}s^{2}+d_{1}s+n_{0}K^{\prime }}$$

(4) $$∆\left(s\right)=1+K_{p}G_{p}\left(s\right)=d_{3}s^{3}+d_{2}s^{2}+d_{1}s+n_{0}K^{\prime }=0$$

According to Table 1, the ultimate gain *K_u_* of the system at critical stability can be expressed as Equation (5), and the auxiliary equation can be expressed as Equation (6). From Equation (6), the oscillation frequency ω_u_ at critical stability can be expressed as Equation (7), and the ultimate period $T_{u}=\frac{2\pi }{\omega_{u}}\left(sec\right)$ can be obtained. By substituting *K_u_* and *T_u_* into Table 2, the proportional constant *K_p_*, integral constant *K_i_*, and differential constant *K_d_* of the controller can be obtained. The *T_i_* and *T_d_* shown in Table 2 are the integral and differential time constants, respectively [29,30,31,32].

(5) $$K_{u}=\frac{d_{2}d_{1}}{d_{3}n_{0}}$$

(6) $$d_{2}s^{2}+n_{0}K^{\prime }=0$$

(7) $$\omega_{u}=\sqrt{\frac{n_{0}K^{\prime }}{d_{2}}}\left(rad/s\right)$$

#### 2.2.2. Design of the PID Controller by Using the Z–N Method

When the system transfer function is unknown, the parameters of the PID controller can be obtained using the Z–N method. In this method, the online adjustment method is employed to obtain the *K_u_* and *T_u_* of the system, and the parameters of the PID controller are obtained according to Table 2.

#### 2.2.3. Evaluation of the ITAE Performance Criterion for the PID Controller

In this study, the ITAE was employed as the performance criterion for evaluating the PID controller [33,34,35,36]. The smaller the ITAE, the more effective the IEG control [37,38]. The ITAE of the system can be expressed as Equation (8) if the system error is *e*(*t*) and the response time is *a*. The ITAE can be expressed as Equation (9) if the Riemann sum is used to approximate Equation (8), where $t_{i}=\frac{a\cdot i}{n}$ and $∆t_{i}=\frac{a}{n}$.

(8) $$\mathrm{ITAE}=\int_{0}^{a}t\left|e\left(t\right)\right|dt$$

(9) $$\mathrm{ITAE}=\underset{n\to \infty }{lim}\overset{n}{\underset{i=1}{\sum }}t_{i}\left|e\left(t_{i}\right)\right|∆t_{i}\;$$

The IEG voltage command in this study is 2 V, the sampling frequency is 1 kHz, and the process time is 120 s. If *v_gap_*(0.001*i*) is the gap voltage measured in 0.001*i* s, the ITAE of the effectiveness of IEG control can be expressed as Equation (10):

(10) $$\mathrm{ITAE}=\overset{120000}{\underset{i=1}{\sum }}\left[\left(0.001i\right)\left|2-v_{gap}\left(0.001i\right)\right|\right]\left(0.001\right)$$

### 2.3. RTMAC System in the Micro-EDM System 

**Figure 4:**
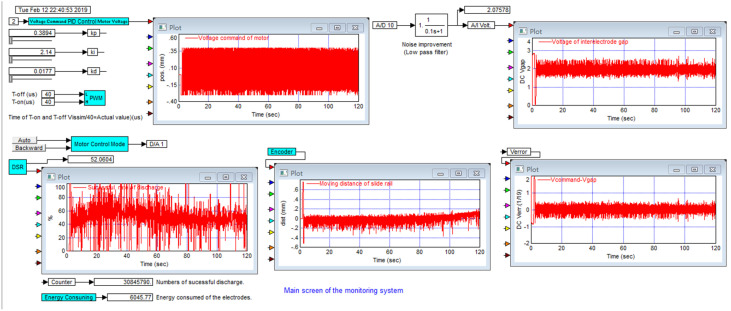
Main screen of the real-time monitoring and control (RTMAC) system.

The functions of the RTMAC system include setting process parameters, providing control commands required by servo circuits, and real-time monitoring and storage of process data [22]. Figure 4 shows the main screen of the system, where the parameters *k_p_*, *k_i_*, *k_d_*, *T_on_*, *T_off_*, and Voltage Command are set according to process requirements. The five subscreens shown in the figure show the motor voltage command, IEG voltage signal, DSR signal, and voltage error *V_error_* between the encoder signal and IEG voltage. The DSR is the ratio of the cumulative number of successful discharges to the total number of discharges. Energy consuming is the cumulative energy consumption within the process time.

**Figure 5:**
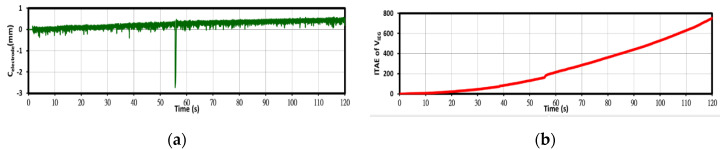
Analysis of process data: (**a**) the C_electrode_ curve and (**b**) the integral of the time-weighted absolute error (ITAE) curve.

The RTMAC system stores process data in a database at a sampling interval of 1 ms, and these data can be used to analyze the process performance. Figure 5 displays the C_electrode_ and ITAE curves obtained from database data. The displacement of the movable electrode can be obtained from the C_electrode_ curve, and the effectiveness of the PID controller can be evaluated using the ITAE curve.

### 2.4. The Settings of the Apparatus

To determine the quality of colloid production, the colloidal absorption spectrum intensity, colloid particle size distribution and suspension, particle size and morphology, and colloidal composition were analyzed using ultraviolet-visible spectrometry (UV-Vis, Thermo-Helios Omega, Thermo Fisher Scientific Inc, Waltham, MA, USA), the Zetasizer nano system (Zetasizer, Nano-ZS90, Malvern Zetasizer, Worcestershire, UK), transmission electron microscopy (TEM, JEM-2100F, JEOL Ltd, Japan), and energy dispersive X-ray spectrometry (EDS, JEM-2100F, JEOL Ltd, Japan), respectively. In UV-Vis, the start and stop wavelength are 190 and 600 nm under the scanning speed and interval of 240 nm/min and 1 nm. In Zetasizer, the light source is He-Ne laser (633 nm), the scattering angle to measure particle size is 90 degrees. The dispersant (DW in this manuscript) setting of the Zetasizer is water with 25 °C in temperature, 0.8872 mPa s in viscosity and 1.330 in refractive index. And the purity of the DW is 7 μS/cm in conductivity and 25 ppm of the dissolved solid. As for TEM, the energy is as high as 200 kV and the magnification is X40,000. And the EDS attached to TEM is used to get composition analysis.

## 3. Results and Discussion

The experiment was conducted under normal temperature which is 25 °C and normal pressure which is 1 atm. The electrode material used in the colloid production was titanium wire (diameter of 1 mm and purity of 99.9%); 150 mL of DW (pH = 6.2) was employed; and the IEG command was 2 V. During the colloid production process, the electrode position was readjusted every 2 min to maintain precise alignment of electrodes; thus, the process time was set to 2 min. Colloid production lasted 20 min and thus involved ten processes. The root locus and Z–N methods were used to identify PID parameters for colloid production, and the ITAE performance criterion was used to evaluate the effectiveness of IEG control in the colloid production process. 

### 3.1. Evaluation of the PID Controller for the Production of TiO_2_ Nanocolloids Designed Using the Root Locus Method

This study used a brushed DC motor (Hitachi 12-19267, Japan) to control the IEG. The results of motor parameter identification experiments revealed that *K_t_* = 0.0250 Nm/A, *K_e_* = 0.0250 V/(rad/s), *J_m_* = 8.523 × 10^−7^ kg·m^2^, and B_m_ = 9.424 × 10^−6^ nm·s/rad. At the rotor position, the armature resistance and inductance were measured using an LCR meter every 18° and 36°. The measurement results revealed that the minimum, average, and maximum values of the armature resistance were 3.45, 7.25, and 9.15 Ω, respectively, whereas the minimum, average, and maximum values of the armature inductance were 1.84, 2.23, and 3.89 mH, respectively. The measured motor parameters were divided into nine combined models, and the PID controller parameters of these models were obtained using the root locus method when designing the PID controller (Table 3).

**Figure 6:**
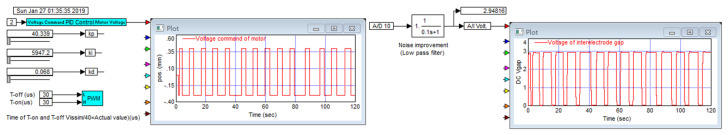
PID-M9 controller motor voltage command and interelectrode gap (IEG) voltage signal in colloid production.

**Figure 7:**
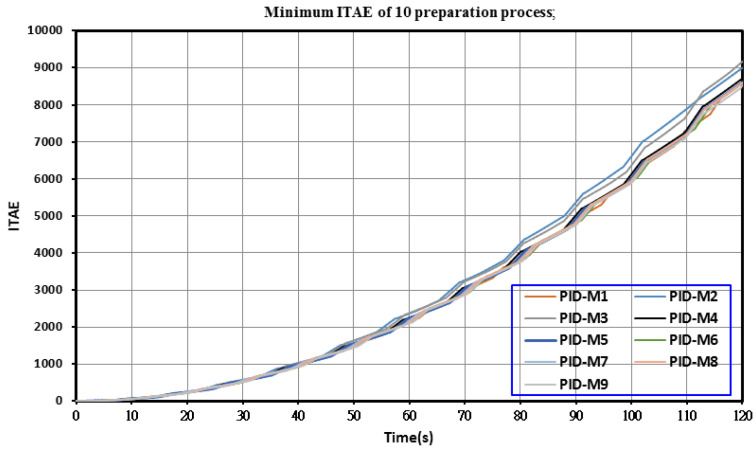
Minimum ITAE values for the production of TiO_2_ nanocolloids by using nine PID controllers designed using the root locus method.

In the case of T_on_–T_off_ of 30–30 µs and a colloid production time of 20 min, the nine PID controllers displayed in Table 3 were used to produce colloids for each model. Figure 6 presents the motor voltage command and IEG voltage signal when the PID-M9 controller was employed to obtain colloids. The amplitude of the IEG voltage was close to 3 V. In all processes for producing the nine types of colloid, the actual value of the IEG voltage was different from the command value (2 V); thus, for these controllers, the ITAE was large in the IEG control. Figure 7 shows the minimum ITAE values (8528.4) for the nine colloids.

**Figure 8:**
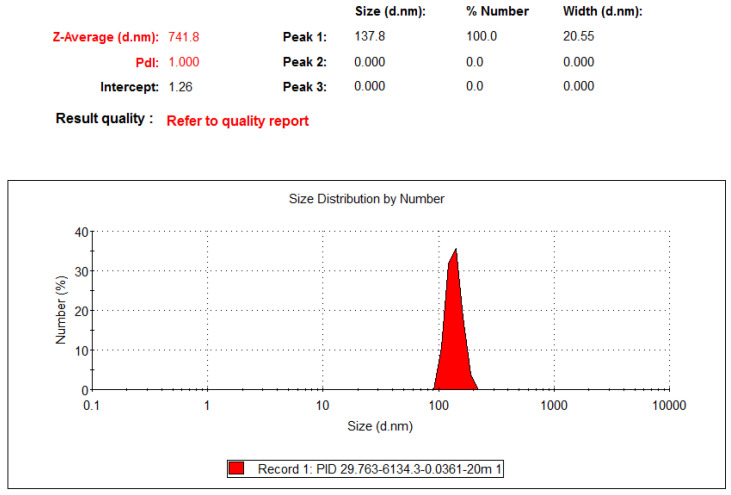
Particle size distribution of TiO_2_ colloids produced by the PID-M1 controller.

The colloids produced using the PID-M1 controller were the smallest, with an average particle size of 741.8 nm, as illustrated in Figure 8. Because the average particle sizes of the nine types of colloids were much larger than 100 nm, none of the PID controllers could produce TiO_2_ NPs.

### 3.2. Evaluation of the PID Controller for the Production of TiO_2_ Nanocolloids Designed Using the Z–N Method

The PID controller designed using the Z–N method was termed the PID-ZN controller, and in its design, *K_p_* was gradually increased after the values of *Kp, Ki*, and *Kd* were set to zero. When *Kp* had increased to 0.649, the IEG voltage began to oscillate periodically with a period of 0.364. The aforementioned *Kp* and period were the system’s *Ku* and *Tu*, respectively. After substituting *Ku* and T_u_ into Table 2, the following controller parameters were obtained: *Kp* = 0.3894, *Ki* = 2.13956 and *Kd* = 0.017718.

**Figure 9:**
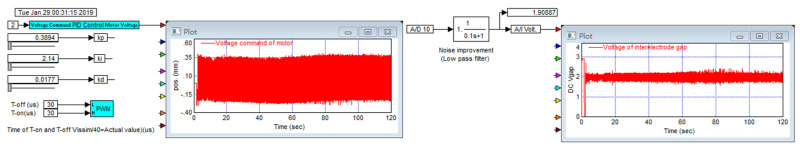
Motor voltage command and IEG voltage signal when the PID–ZN controller was employed to produce colloids.

**Figure 10:**
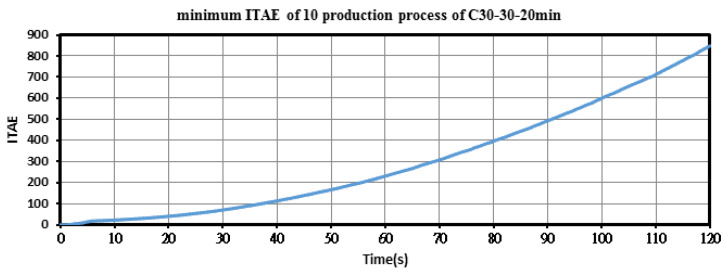
Minimum ITAE values for the PID–ZN controller when producing C30-30-20 min colloids.

TiO_2_ colloids produced using the PID-ZN controller with T_on_–T_off_ of 30–30 µs and a colloid production time of 20 min were named C30-30-20 min colloids. Figure 9 displays the motor voltage command and IEG voltage signal during production of C30-30-20 min colloids. The IEG voltage was maintained at the command value of 2 V. The maximum and average values of the ITAE during C30-30-20 min colloid production were 1137.1 and 988.74, respectively. Figure 10 shows the minimum ITAE for production of C30-30-20 min colloids.

**Figure 11:**
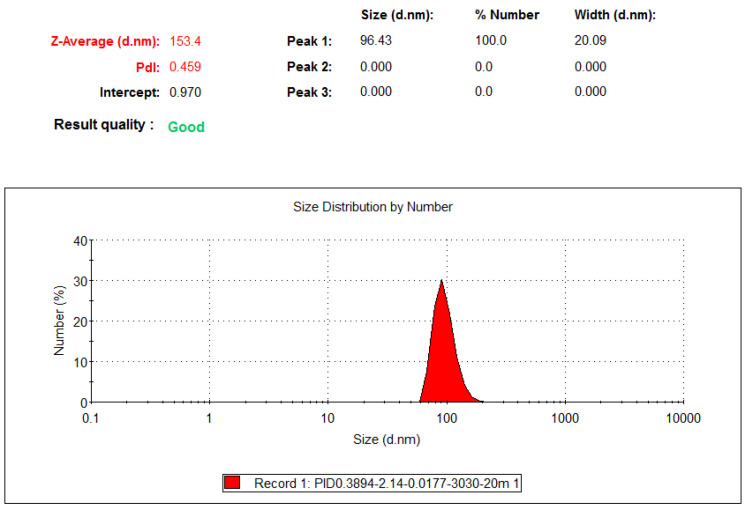
Particle size distribution for C30-30-20 min colloids.

The particle size distribution of C30-30-20 min colloids is illustrated in Figure 11. The average particle size was 153.4 nm in diameter, and the colloids contained a considerable number of NPs. This result reveals that TiO_2_ nanocolloids could be produced using the PID-ZN controller.

This study employed the PID–ZN controller, eight pulse periods (10–10, 20–20, 30–30, 40–40, 50–50, 60–60, 80–80, and 100–100 µs), and eight colloidal production durations (10, 20, 30, 40, 50, 60, 80, and 100 min) to produce TiO_2_ colloids. The colloidal production duration was set to 30 min, and we increased the pulse periods from 10–10 to 100–100 µs. The maximum and minimum absorbance values were 0.344 and 0.074, respectively. In addition, the maximum absolute value of ζ potential was −40.2 mV and the minimum absolute value of ζ potential was −22.0 mV. Among the eight preparation conditions, the pulse periods of 40–40 µs had an absorption of 0.344 and a ζ potential of −40.2 mV. Therefore, the number and suspension of NPs in the colloid prepared using pulse periods of 40–40 µs were preferable to those of the colloids prepared under other conditions. The aforementioned analysis of the pulse period indicates that the absorption of colloids prepared in the first four pulse periods increased with period length, meaning that the T_off_ time of these four processes is sufficient for restoring the insulation of the electrode gap. The absorption of colloids prepared in the last four pulse periods decreases as period length increases. For these four processes, the T_on_ time was too long for the T_off_ time to restore the insulation of the electrode gap. When the pulse periods were set to 40–40 µs, the analysis of the eight colloidal production durations revealed that absorption increases with colloid preparation time, indicating that the colloid did not reach saturation within 100 min.

**Figure 12:**
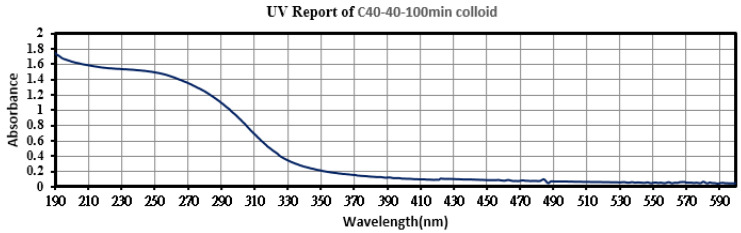
Absorption spectrum of C40-40-100 min colloids.

**Figure 13:**
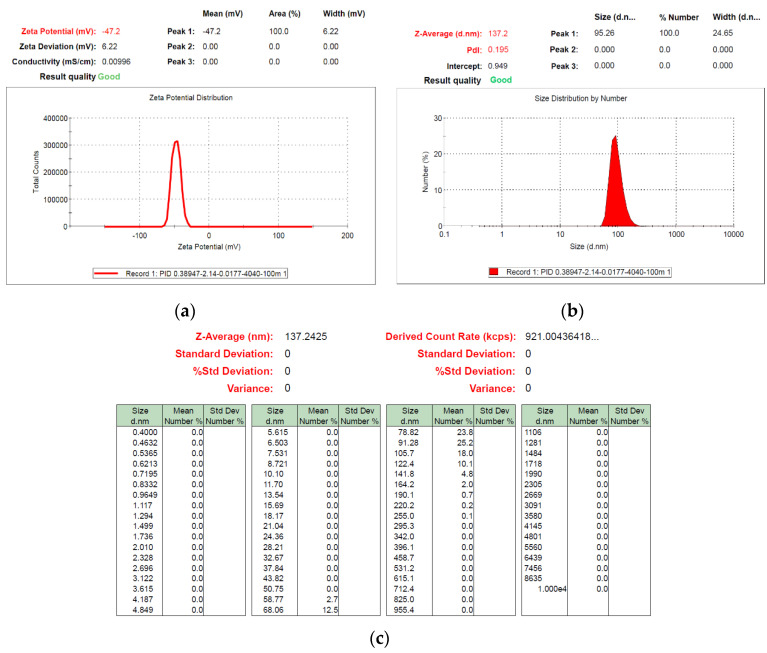
Analysis of C40-40-100 min colloids: (**a**) ζ potential; (**b**) particle size distribution; and (**c**) particle size statistics report by number of colloids.

**Figure 14:**
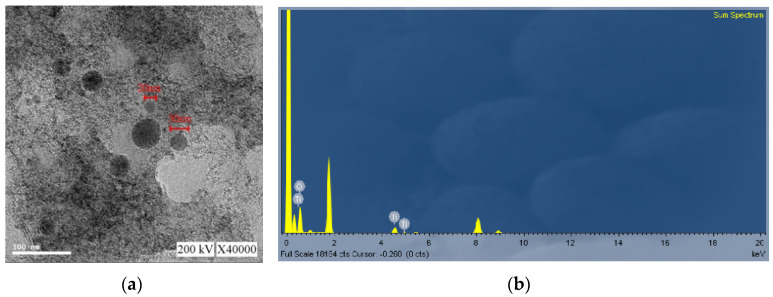
Analysis of C40-40-100 min colloids: (**a**) enlarged TEM image at 40,000× magnification; (**b**) EDS composition analysis.

The colloids produced using a 40–40 µs pulse period and colloid production time of 100 min were labeled the C40-40-100 min colloids. Among the TiO_2_ colloids prepared in the eight pulse periods and eight colloidal production durations, the C40-40-100 min colloids had optimal absorption, suspension, and number of NP’s. The C40-40-100 min colloids were analyzed using UV-Vis and the Zetasizer; the findings are presented in Figure 12 and Figure 13. The colloids had a wavelength of 245 nm, absorption of 1.511, ζ potential of −47.2 mV, and average particle size of 137.2 nm in diameter; 64.2% of particles were smaller than 91.28 nm. Figure 14a,b present a TEM image and the EDS composition analysis of the C40-40-100 min colloids, respectively. The colloidal particles were spherical, and the minimum particle size was approximately 20 nm in diameter. The colloids were composed of only Ti and O. Compared with the C30-30-20 min colloids, the C40-40-100 min colloids were smaller and more numerous and had more satisfactory suspension properties.

## 4. Conclusions

The monitoring and control function of the micro-EDM system developed in this study contributed to the high process efficiency. When TiO_2_ nanocolloids were produced using the PID–ZN controller with T_on_–T_off_ of 40–40 µs and a colloid production time of 100 min, the absorbance of the produced TiO_2_ nanocolloids was 1.511, the ζ potential was −47.2 mV, the average diameter was 137.2 nm, and the percentage of particles smaller than 91.28 nm was 64.2%. TEM revealed that the colloidal particles were spherical. The characteristics of colloids confirmed that the proposed system can produce TiO_2_ nanocolloids with excellent suspension stability. In this study, TiO_2_ nanocolloids were produced and collected in DW; thus, escape of nanoparticles did not occur in the process environment. The results of this study are as follows:

1. The micro-EDM system successfully produced TiO_2_ nanocolloids with excellent suspension stability under room temperature and atmospheric pressure, and colloids contained no elements other than Ti and O. Their application in environmental pollution prevention would help prevent secondary pollution to the environment.
2. Compared with industrial-grade electrical discharge machines, the micro-EDM system has the advantages of small size and low cost. These factors can enhance the advantages of the ESDM in the production of TiO_2_ NPs.
3. The parameters of the PID controller designed using the Z–N method (*K_p_* = 0.3894, *K_i_* = 2.13956 and *K_d_* = 0.017718) were obtained through the online adjustment method. This method considers the electrode material and characteristics of the dielectric fluid. Therefore, the controller‘s efficiency at controlling the IEG was superior to that exerted by the PID controller designed using the root locus method.

## Figures and Tables

**Table 1:** Routh table established according to Equation (4).

$s^{3}$	$d_{3}$	$d_{1}$
$s^{2}$	$d_{2}$	$n_{0}K^{\prime }$
$s^{1}$	$\frac{d_{2}d_{1}-d_{3}n_{0}K^{\prime }}{d_{2}}$	
$s^{0}$	$n_{0}K^{\prime }$	

**Table 2:** Z–N tuning rules for the proportional–integral–derivative (PID) controller.

$K_{p}$	$T_{i}$	$T_{d}$	$K_{i}$	$K_{d}$
0.6$K_{u}$	$T_{u}$/2	$T_{u}$/8	$K_{p}/T_{i}$	$K_{p}\times T_{d}$

**Table 3:** Determination of PID controller parameters for the DC motor by using the root locus method.

Mode Type	PID Controller	Ra (Ω)	La (mH)	$K_{p}$	$K_{i}$	$K_{d}$
Mode 1	PID-M1	3.45	1.84	29.763	6134.3	0.0361
Mode 2	PID-M2	3.45	2.23	24.588	4603.4	0.0328
Mode 3	PID-M3	3.45	3.89	14.170	2008.6	0.025
Mode 4	PID-M4	7.25	1.84	65.748	13,915	0.0777
Mode 5	PID-M5	7.25	2.23	54.282	10,436	0.0706
Mode 6	PID-M6	7.25	3.89	31.196	4541	0.0536
Mode 7	PID-M7	9.15	1.84	85.072	18,236	0.0992
Mode 8	PID-M8	9.15	2.23	70.227	13,675	0.0902
Mode 9	PID-M9	9.15	3.89	40.339	5947.2	0.068
